# Pregnancy and Neonatal Diabetes Outcomes in Remote Australia (PANDORA) study

**DOI:** 10.1186/1471-2393-13-221

**Published:** 2013-12-01

**Authors:** Louise J Maple-Brown, Alex Brown, I-Lynn Lee, Christine Connors, Jeremy Oats, Harold D McIntyre, Cherie Whitbread, Elizabeth Moore, Danielle Longmore, Glynis Dent, Sumaria Corpus, Marie Kirkwood, Stacey Svenson, Paula van Dokkum, Sridhar Chitturi, Sujatha Thomas, Sandra Eades, Monique Stone, Mark Harris, Chrissie Inglis, Karen Dempsey, Michelle Dowden, Michael Lynch, Jacqueline Boyle, Sue Sayers, Jonathan Shaw, Paul Zimmet, Kerin O’Dea

**Affiliations:** 1 Menzies School of Health Research, Charles Darwin University, PO Box 41096, Casuarina NT 0811, Darwin, Australia; 2 Division of Medicine, Royal Darwin Hospital, Darwin, Australia; 3 South Australian Health and Medical Research Institute, Adelaide, Australia; 4 School of Population Health, University of South Australia, Adelaide, Australia; 5 Northern Territory Department of Health, Darwin, Australia; 6 Melbourne School of Population and Global Health, University of Melbourne, Melbourne, Australia; 7 Mater Research Institute, University of Queensland, Brisbane, Australia; 8 Aboriginal Medical Services Alliance Northern Territory, Darwin, Australia; 9 Alice Springs Hospital, Northern Territory, Alice Springs, Australia; 10 Baker IDI Heart and Diabetes Institute, Central Australia, Australia; 11 Department of Obstetrics and Gynaecology, Royal Darwin Hospital, Darwin, Australia; 12 Sydney Medical School, The University of Sydney, Sydney, Australia; 13 Department of Paediatrics, Royal Darwin Hospital, Darwin, Australia; 14 Mater Children’s Hospital, South Brisbane, QLD, Australia; 15 Healthy Living NT, Darwin, Australia; 16 Aboriginal Health Council of West Australia, Perth, Australia; 17 Pathology Network, Top End Health and Hospital Services, Darwin, Australia; 18 Monash Centre for Health Research and Implementation, School of Public Health and Preventive Medicine, Monash University, Melbourne, Australia; 19 Baker IDI Heart and Diabetes Institute, Melbourne, Australia

**Keywords:** Diabetes in pregnancy, Gestational diabetes, Antenatal care, Birth weight, Neonatal body composition, Anthropometry, Indigenous Australian, Aboriginal

## Abstract

**Background:**

Diabetes in pregnancy carries an increased risk of adverse pregnancy outcomes for both the mother and foetus, but it also provides an excellent early opportunity for intervention in the life course for both mother and baby. In the context of the escalating epidemic of chronic diseases among Indigenous Australians, it is vital that this risk is reduced as early as possible in the life course of the individual. The aims of the PANDORA Study are to: (i) accurately assess rates of diabetes in pregnancy in the Northern Territory (NT) of Australia, where 38% of babies are born to Indigenous mothers; (ii) assess demographic, clinical, biochemical, anthropometric, socioeconomic and early life development factors that may contribute to key maternal and neonatal birth outcomes associated with diabetes in pregnancy; and (iii) monitor relevant post-partum clinical outcomes for both the mothers and their babies.

**Methods/Design:**

Eligible participants are all NT women with diabetes in pregnancy aged 16 years and over. Information collected includes: standard antenatal clinical information, diagnosis and management of diabetes in pregnancy, socio-economic status, standard clinical birth information (delivery, gestational age, birth weight, adverse antenatal and birth outcomes). Cord blood is collected at the time of delivery and detailed neonatal anthropometric measurements performed within 72 hours of birth. Information will also be collected regarding maternal post-partum glucose tolerance and cardio-metabolic risk factor status, breastfeeding and growth of the baby up to 2 years post-partum in the first instance.

**Discussion:**

This study will accurately document rates and outcomes of diabetes in pregnancy in the NT of Australia, including the high-risk Indigenous Australian population. The results of this study should contribute to policy and clinical guidelines with the goal of reducing the future risk of obesity and diabetes in both mothers and their offspring.

## Background

The antecedents of non-communicable diseases are early in life, beginning *in utero*. In order to address the escalating epidemic of chronic diseases among Indigenous Australians [1], we need to reduce risk as early as possible in the life of an individual. Diabetes in pregnancy (DIP) is associated with increased risk of adverse pregnancy outcomes for both the mother and the child, but also provides an early opportunity for intervention in the life of both mother and baby.

Diabetes in pregnancy includes gestational diabetes mellitus (GDM) and pre-existing diabetes (type 1 and type 2) in pregnancy. Rates of both pre-existing diabetes in pregnancy and GDM are higher in Indigenous Australian women compared to the general Australian population despite a younger age of giving birth [2,3]. Particularly striking are the high rates of pre-existing type 2 diabetes in pregnancy among Indigenous Australian women, with rates over ten times greater than those reported in the general Australian population [4]. The recent increase in T2DM in pregnancy among Indigenous Australian women is of concern as it is associated with significantly higher risk (compared to GDM) for outcomes such as stillbirth and congenital malformations [3,5-7].

Large international studies have reported the adverse perinatal and obstetric outcomes associated with hyperglycaemia in pregnancy [8] and the beneficial perinatal outcomes that result from good blood glucose control in pregnancy, including lower rates of babies born large for gestational age, pre-eclampsia, shoulder dystocia and birth trauma [9]. Diabetes in pregnancy has also been associated with increased future risk of other chronic diseases, for both the mother and baby. Future risk of type 2 diabetes in the mother is increased seven-fold following a pregnancy complicated by GDM [10]. Canadian data report very high future risk for Indigenous women: over 70% of women developed type 2 diabetes within 4 years of GDM diagnosis [11]. Children of mothers with DIP are more likely to be obese in adolescence [12], and have diabetes in early adulthood, with the rates of type 2 diabetes substantially higher in offspring of mothers with pre-existing DIP than those of mothers with GDM or without DIP [13].

We have developed a partnership between researchers, health care providers and policy organisations in the Northern Territory (NT), Australia, to address the issue of DIP in the high-risk population of the NT. The partnership includes a detailed research component: Pregnancy And Neonatal Diabetes Outcomes in Remote Australia – The PANDORA Study. The NT covers a large geographical area (1.35 million square kilometres), but has a relatively small population (230,000), with a population density of 0.2 people/km^2^[14]. From 2001 to 2005, the average annual number of births in the NT was 3566, of whom 38% were born to Indigenous mothers [15].

### Aims

The aims of The PANDORA Study are:

1) To accurately assess the rates of diabetes in pregnancy in the Northern Territory, Australia.

2) To report demographic, clinical, biochemical, anthropometric and socioeconomic factors that may contribute to key maternal and neonatal outcomes associated with DIP in the NT.

3) To monitor relevant clinical outcomes for both the mothers and their babies, and provide reliable information around future health risk for the NT.

## Methods/Design

### Study design and overview

This study will be the research arm of the larger NT DIP Partnership Program. The NT DIP Partnership aims to: (i) improve systems and service delivery for all women in the NT with DIP, in order to improve outcomes for mother and baby; (ii) reduce the gap between evidence and practice in relation to screening, management and post-partum follow-up of women with DIP and their baby; (iii) establish systems that enable close monitoring of relevant clinical outcomes for mothers and babies. These aims will be achieved by: (i) establishing an NT DIP clinical register; (ii) developing and evaluating an appropriate model of care for DIP; (iii) instituting early universal detection of DIP; (iv) enhancing capacity of health care professionals in identifying and managing DIP; and (v) delivering a detailed research component to accurately assess antenatal characteristics and neonatal outcomes of DIP. The research component is The PANDORA Study and is described here.

### Setting & location

Participants will be residents of the Northern Territory, Australia. Participation in the study is facilitated through the following hospitals, Royal Darwin Hospital, Katherine District Hospital, Gove District Hospital, Alice Springs Hospital, Tennant Creek Hospital; Aboriginal Medical Services, Danila Dilba Health Services, Bagot Community Health Centre, Wurli Wurlinjang, Sunrise Health Service Aboriginal Cooperation, Miwatj Health Aboriginal Corporation, Central Australian Aboriginal Congress, Anyinginyi Health Aboriginal Corporation, Pintubi Homelands, Ampilatawatja Health Care Centre, Nganampa Health Council, Amoonguna; and health facilities, Healthy Living NT and NT Department of Health remote. Consultation with other Aboriginal Medical Services in the NT regarding their participation in the study is ongoing.

### Participants & recruitment

Potential participants will be all NT women (Indigenous and non-Indigenous) with any type of diabetes in pregnancy (type 1 diabetes mellitus, type 2 diabetes mellitus and gestational diabetes mellitus). Participants are aged 16 years and above, from urban, rural and remote regions of the NT. A NT DIP Clinical Register was established in 2011–2012 as part of the larger NT DIP Partnership Program. Women with DIP from the NT are referred to the register and information maintained for clinical use by both specialist and primary health care teams. The register data are collected primarily by the senior diabetes nurse educators at Royal Darwin Hospital and Alice Springs Hospital, with additional referrals received from diabetes nurse educators at other sites, including Healthy Living NT, Katherine District Hospital and Gove District Hospital. Once the woman is in contact with either the specialist team or the research team, they are invited to participate in the research study (PANDORA).

### Funding

Major funding is provided by the National Health and Medical Research Council of Australia (NHMRC Partnership Project Grant #1032116). Additional support (including pilot funding) was received from NHMRC Program Grant #631947.

### Ethics

The study is approved by the joint Menzies School of Health Research – Northern Territory Department of Health Human Research Ethics Committee, including the Aboriginal sub-committee. The study is also approved by the Central Australian Human Research Ethics Committee.

### Staff

Six research staff are employed over the course of the study. Two post-graduate research students are participating in the study and are substantially involved in data collection. All research staff members have prior health qualifications and experience: all are registered nurses, of whom two are also diabetes educators, and four are midwives. The post-graduate research students are a specialist endocrinologist and a paediatric endocrinologist.

The Partnership involves considerable in-kind contribution from all partners: NT Department of Health, AMSANT, Baker IDI and Healthy Living NT (primarily health professional staff time and travel costs).

### Consent

Potential participants have face-to-face discussion with a study staff member, are provided with visually enhanced written information about the study and given an opportunity to ask questions. An interpreter is employed to explain study information when required. Those who indicate interest in participating are then asked to complete written consent. Consent is obtained using the NHMRC Guidelines for Ethical Conduct in Aboriginal and Torres Strait Islander Health Research. All participants are informed that their participation is voluntary, that they can refuse or withdraw from participating, they need give no reason or justification for their decision to withdraw, and that it would not affect their medical care. A parent or guardian is asked to sign the form in addition to the participant if the participant is under 18 years old. Participants are asked to give separate consent for various elements of the study, including cord blood collection, baby body measurements, questionnaire and collecting further information from their medical records. They are informed that their cord blood samples will be stored at Menzies School of Health Research for 25 years and asked whether their cord blood samples could be used for future studies related to diabetes and cardiovascular disease.

### Screening criteria for gestational diabetes

The diagnosis of GDM is based on the Australian Diabetes in Pregnancy Society (ADIPS) guidelines, which changed during the course of The PANDORA Study. Prior to 2012, guidelines were that all pregnant women be screened routinely for GDM at 24–28 weeks gestation with a 50 g glucose challenge test (GCT). A positive cut-off value after 1 hr in GCT was 7.8 mmol/L plasma glucose for the 50 g load. Women with a positive GCT proceeded to have a fasting 75 g oral glucose tolerance test (OGTT) for which the diagnostic criteria was one or more of the following values: fasting glucose ≥ 5.5 mmol/L or 2-h plasma glucose ≥ 8.0 mmol/L [16]. From 2012, GDM was diagnosed based on new ADIPS recommendations [17] of universal OGTT (without an initial GCT), with an increase in implementation of these guidelines towards the end of 2013. From the end of 2013, GDM was diagnosed by one or more of the following values from the 75gm OGTT: fasting glucose ≥ 5.1 mmol/L, 1-h plasma glucose ≥ 10.0 mmol/L or 2-h plasma glucose ≥ 8.5 mmol/L [17]. There was an overlap period for approximately 2 years following the introduction of the new guidelines, during which time women were diagnosed with GDM by either of the above guidelines. Local guidelines (Minymaku Kutju Tjukurpa Women’s Business Manual 4th edition [18]) are used in conjunction with the above guidelines and recommend HbA1c and random glucose at first antenatal visit in women at high risk of undiagnosed type 2 diabetes (all Indigenous Australian women). Consideration for early 75gm OGTT in these high risk women was recommended in the NT according to IADPSG guidelines [19]. Women with a clinical diagnosis of likely undiagnosed type 2 diabetes, first diagnosed in pregnancy, are included in the group of women classified as GDM for the purposes of analysis. A clinical diagnosis of likely undiagnosed type 2 diabetes was based on one of the following at first antenatal visit: fasting glucose ≥7.0 mmol/l, 2 hour glucose ≥11.1 mmol/l, HbA1c ≥6.5% [19].

### Data collection techniques

Once written consent is attained for the research study, the relevant clinical information for participants is transferred from the clinical register to the research study database. The following information is collected:

(i) Antenatal data collected by the clinical team:

a. Assessment of antenatal health: weight (in first and late third trimester), height, blood pressure, gravidity and parity, oral glucose tolerance test, HbA1c (when done), haemoglobin, serum creatinine, urine albumin-creatinine ratio, type of diabetes in pregnancy (GDM, type 1 diabetes, type 2 diabetes, or likely pre-existing type 2 diabetes), estimated date of delivery (ultrasound)

b. Additional standard antenatal care data is collected by the clinical team, and collated peri-partum by the research team: management of DIP (oral agents, use and type of insulin), other medications, antenatal incidence of maternal hypoglycaemia (mild/severe), hospitalisation before delivery, other comorbidities, obstetric antenatal complications (e.g. antepartum haemorrhage, placenta praevia), antenatal mode of care and specialist involvement, vitamin D status, physical activity, dietary habits and Edinburgh depression scale score [20].

(ii) Further antenatal data collected by the research team:

a. Assessment of socio-economic status and related personal details by questionnaire: age, contact details, hospital record number, maternal and paternal ethnicity, first language, language spoken at home, ethnicity of maternal and paternal grandparents for Aboriginal and Torres Strait Islander participants, income source, ever worked, employment status, education level, housing tenure, landlord type (government, private, other), self-reported pre-pregnancy weight, pre-conception medication and pre-conception care.

b. Medical history and medications- self report and from medical records (where available) including assessment of cigarette smoking, use of pituri (chewing of wild tobacco plants), tobacco chewing, alcohol and other recreational drugs used during pregnancy, past medical history and supplements/medications during pregnancy, maternal and paternal family history of diabetes.

c. Maternal obstetric information: hypertension, pre-eclampsia, eclampsia, ultrasound scan of morphology, number of ultrasound scans for growth assessment.

d. Additional questionnaires to assess stress and depression were performed in a sub-study of participants: Patient Health Questionnaire 9 (PHQ9) and adapted PHQ9 [21]

(iii) Postnatal data collected by the research team

a. Neonatal information: location, birth date and time, birth outcome, time to first breastfeed, APGAR Score, shoulder dystocia, neonatal obstetric trauma, severe adverse event (death, delivery <32 weeks, prolonged hospitalisation with >1 week special care nursery, condition which results in significant disability/incapacity or requires medical intervention to prevent permanent damage), major and minor congenital malformation, admission and length of stay to special care nursery, neonatal hypoglycaemia, hyperbilirubinaemia, phototherapy, exchange transfusion, hypoxic ischaemic encephalopathy, neonatal hypocalcaemia, meconium aspiration, respiratory distress, gestational age at delivery. Congenital malformations defined as: structural abnormalities detected prior to birth by ultrasound or at or soon after birth by clinical examination, X-ray, or ultrasound and, in the event of neonatal or perinatal death, by autopsy; and classified as major if fatal, potentially life threatening, likely to lead to serious handicap, or likely to lead to major cosmetic defect.

b. Maternal obstetric information: mode of delivery, labour complications (type of instrumental delivery, third/fourth degree perineal tear, postpartum haemorrhage, uterine rupture, foetal distress, cord prolapse, manual removal of placenta, meconium stained liquid and others), need and reason for induction of labour, indication for caesarean or operative vaginal delivery.

c. At discharge: breastfeeding at discharge, type of contraception planned, medications.

(iv) Neonatal anthropometric measurements performed by the research team: neonates have anthropometric measurements completed within 72 hours of birth. If measurements are unable to be performed within 72 hours of birth, they may be performed up to 120 hours of birth and recorded as such. Neonatal anthropometric measurements are conducted with a calibrated scale for weight (Darwin: Ultracare electronic scales, Alice Springs: Nuweigh, Australia), a measuring board for length (Seca, Hamburg, Germany), Holtain calipers (Holtain Ltd, Crosswell, Pembrokeshire, United Kindgom) for skin fold measurements, a measuring tape for circumferences, and a metal anthropometer (Scientific Martin Pelvimeter) for limb length. All limb and skin fold measures are made on the left side, except when the left limb is unavailable for access (eg the neonate had intravenous therapy in the left arm). For all measurements, the mean of the two measurements was used, unless a third measurement was taken (if the two measures differed by more than the prescribed amount). When a third measurement was taken, if two of three measurements differed by less than the pre-specified amount, the mean of those two was used, otherwise the mean of all three was used [22].

a. A bare weight is obtained on a calibrated scale, and recorded to the nearest 5 gram. This procedure is repeated and recorded twice. A third measurement is performed if the first two measurements are not within 10 grams of each other.

b. Length is obtained on a standardized plastic length board and recorded in centimetres. The infant is positioned on its back so that the vertebrae touch the base of the length board. The crown of the head lies against the vertical end of the length board. Keeping the infant’s head in the proper position, the examiner uses the thumb of his/her left hand and firmly presses the infant’s chest into the base of the length board to ensure that the spine is straight. The examiner then uses his/her right hand to extend the legs straight. When the infant’s body is positioned properly, the examiner measures the length with the movable foot board attached to the length board. Lengths are obtained and recorded to the nearest 0.1 cm at least twice. If lengths differ by more than 0.5 cm, a third length is taken and recorded.

c. Circumference of the head is measured with a measuring tape, including the occipital protuberance, measured while the baby is quiet.

d. Skinfold thickness assessment is performed at 3 sites. Study staff were trained by a Clinical and Data Coordinating Centre Staff member of the Hyperglycaemia and Adverse Pregnancy Outcomes (HAPO) Study, using the HAPO video and methods [22,23]. The skin fold site is marked using a felt-tip pen and each site is identified as follows: the triceps skin fold is measured over the triceps, midway between the acromion and olecranon. The subscapular skin fold is measured at the lower angle of the scapula. The flank skin fold is measured in the mid-axillary line just above the crest of the ilium. The skin fold is obtained by lifting the top layer of the skin at the specific landmark with the thumb and the index finger. While holding onto the skin, the calipers are applied perpendicular to the fold, on the site marked. While maintaining the grasp of the skinfold, the calipers are released so that full tension is placed on the skinfold. The dial is read to the nearest 0.1 mm, 30–45 seconds after the grip is fully released, or until the reading has stabilized. Two readings are taken at each site. A third reading is done if the two readings differ by more than 0.5 mm.

e. The following additional body composition measures are performed in a detailed body-composition sub-study of the larger PANDORA Study:

– Skin fold thickness assessment is performed at 2 additional sites: thigh and abdomen [24]. The thigh skin fold is measured midway between the crural fold and the large semilunar crease above the patella when the leg is fully extended in the longitudinal plane of the leg. The abdomen skin fold is measured midway between the xiphoid and the umbilicus on the nipple line.

– Circumferences of the chest (across the nipple line), abdomen at the liver and umbilicus, and mid-portion of the upper and lower extremities are measured with a measuring tape.

– Lengths of the upper and lower limbs are determined with a metal anthropometer.

(v) Cord blood collection:

A specimen of cord blood is obtained from all neonates born to the study participants for analysis of glucose, C-peptide, lipids, C-reactive protein, interleukin-6, adiponectin and leptin. The specimen is obtained by free drainage (free flow) of cord blood or drawn by needle aspiration (puncture) from a clamped segment of an umbilical vein. The specimen is stored in a foam cooler for transport to the hospital laboratory.

(vi) Maternal follow-up information collected by research team:

Six week post partum oral glucose tolerance test results for women with gestational diabetes, rates and duration of breast feeding, assessment at 24 months of metabolic and cardiovascular risk (adult health check), use and mode of contraception, cigarette smoking, weight, medications, subsequent pregnancy.

(vii) Follow up of baby:

Any fatal event, identification of congenital abnormalities beyond birth, significant medical diagnoses, hospitalisation, growth at 1, 2,4, 6–8 and 15–18 months in the first instance. Additional funding will be sought for follow-up of the baby and mother beyond that detailed above.

### Laboratory methods

Collected cord blood samples are centrifuged for 10 minutes at 3000 revolutions per minute within 1–2 hours of collection. The time between collection and centrifugation is noted for all cord blood samples. Following centrifugation, plasma is divided from the blood tube ensuring no cells are transferred to sample vials.

Vials are transported on ice for storage in -70°C freezers. Analysis of glucose and lipids are performed at each centre as outlined in Table 1. Samples are stored for analysis at a later date for: c-peptide, C-reactive protein, interleukin-6, adiponectin and leptin.

**Table 1:** Analysis of glucose and lipids at each centre

	**Laboratory**	
Test	Royal Darwin Hospital (and Darwin Private Hospital)	Alice Springs Hospital
Lipids	Ortho-Clinical Diagnostics, Fusion 5.1	Ortho-Clinical Diagnostics, Fusion 5.1
Glucose	Ortho-Clinical Diagnostics, Fusion 5.1	Ortho-Clinical Diagnostics, Fusion 5.1

### Sample size

All NT women with DIP, estimated to be 250–300 women/year, are eligible for the study with the exception of those aged <16 years. We expect that approximately 60% of these women will consent to the research study. Consent of participants for PANDORA commenced in November 2011 and is expected to continue until 2015. The estimated number of participants is at least 700 women.

### Data handling & statistical methods

At the time of data collection, data is collected on paper forms and then entered into a Microsoft Access database. Data from each site is stored securely at Menzies School of Health Research (Darwin) or Baker IDI (Alice Springs). All statistical analysis will be performed using the latest available release of Stata software (Stata Corporation, College Station, TX). Rates of diabetes in pregnancy (in NT and for Indigenous babies in NT) will be calculated using the denominators of all NT births and all births in the NT with Indigenous mothers (using NT Department of Health Midwives’ Collection birth data). Analysis will be performed for summary statistics of rates of diabetes in pregnancy (known pre-existing, undiagnosed pre-existing and gestational diabetes); antenatal characteristics of women with DIP (age, geographic region, gravida/parity etc.); clinical management of DIP (use of insulin, oral medications, maternal hypoglycaemia). Bivariate and multivariate associations will be determined for demographic, clinical (including neonatal anthropometric measurements), biochemical (including cord blood) and socioeconomic variables with the following key neonatal and maternal outcome variables: birth weight, gestational age, large for gestational age, neonatal hyperinsulinaemia (cord blood C-peptide >90th centile), neonatal adiposity (fat for gestational age > 90th centile), pre-eclampsia, birth injury/complication, adverse labour events, delivery mode and breastfeeding rates (discharge, 6–8 weeks and 7–9 months). Analyses will be performed for the whole group and stratified by ethnic group. After determining bivariate associations between variables (using Spearman correlations for continuous variables and two-tailed t-tests for dichotomous variables), risk factors will be selected for entry into logistic regression models using the backward selection method, the outcome variables being the end-points outlined above.

## Discussion

The aims of the NT Diabetes in Pregnancy Partnership are to improve clinical care and outcomes of DIP in the NT, and to improve future health outcomes for both the mother and her baby through an integrated, multi-faceted approach. This includes a detailed research component, outlined above: the PANDORA Study. This study should provide answers to three key questions: (i) rates of diabetes in pregnancy in the NT, including the high risk Indigenous Australian population of remote and urban NT; (ii) demographic, clinical, biochemical, anthropometric and socioeconomic factors that may contribute to key maternal and neonatal outcomes associated with diabetes in pregnancy; and (iii) relevant clinical outcomes for mothers with DIP and their babies. The results of this study should contribute to policy and clinical practice guidelines of management of DIP and follow-up of mother and baby.

The study includes detailed neonatal anthropometric measurements performed using skinfold measurements, limb lengths and body circumferences in order to accurately assess neonatal body fat. This method has been well validated and is reproducible [24,25] with regular training of research staff. It is notable that although rates of low birth weight are higher among Indigenous Australian babies than the general Australian population (without DIP), there is increasing evidence that rates of low birth weight are reducing among Indigenous Australian babies [26]. In this context, assessment of foetal adiposity and its contribution to birth weight is important in this high risk population. Birth weight alone is not a sensitive marker of foetal overgrowth in diabetes in pregnancy [25], and detailed assessment of foetal adiposity is required in order to assess if hyperglycaemia in pregnancy could be contributing to a phenotype previously described in India, that of a “thin-fat baby” [27]. Thus the detailed assessment of neonatal body fat and cord blood biochemistry in this study should provide evidence for clinical guidelines in a field where evidence is currently lacking: the role of neonatal body composition (relative contributions of adipose tissue mass and fat free mass to birth weight) in a population at high risk of both low birth weight (from factors such as malnutrition, maternal smoking) and high birth weight/large for gestational age due to hyperglycaemia in pregnancy.

The study is likely to also inform the design of future work to reduce risk of future obesity, diabetes and cardiovascular disease in both the mothers and babies. Although better glycaemic management of pregnant women has been shown to reduce the rate of maternal and neonatal adverse outcomes in the short term [28]; to date there is no evidence for reduction in the future risk of diabetes, cardiovascular disease in the mother or of obesity and type 2 diabetes in the child, by glycaemic intervention in pregnancy. Observational studies have reported higher rates of diabetes in children of mothers with diabetes in pregnancy, and thus reduced levels of glucose in pregnancy would appear to influence the child’s future risk of diabetes [13,29,30]. Despite substantial reduction in the risk of macrosomia at birth in the interventional arm of the Australian Carbohydrate Intolerance Study in Pregnant Women [9], there was no difference between the intervention and routine-care groups in the child’s body mass index at 4–5 years old [31]. A recent Danish study of non-diabetic adult offspring born to women with diabetes in pregnancy (type 1 diabetes and GDM) showed reduced insulin sensitivity and impaired beta cell function [32]. The current study is designed for ongoing longitudinal follow-up of both the mothers and babies, and additional funding will be sought for follow-up.

In conclusion, the NT Diabetes in Pregnancy Partnership and PANDORA Study will have significance for policy and planning in the NT, as well as in other urban, rural and remote regions of Australia. Efforts to reduce risk as early as possible in the life course are crucial in order to reduce the gap between Indigenous and non-Indigenous health outcomes in Australia.

## Competing interests

All authors declare that they have no competing interests.

## Authors’ contributions

LMB drove the design of the study protocol for funding and ethics applications, coordinated data collection and data management, and drafted the manuscript. LMB and AB initiated the study concept, design and partnership. AB, CC, JO, HDM, EM, JS, PZ, KOD provided important intellectual input into the study design, funding application and revision of the manuscript. CW, MK and SS contributed to project management, data collection, data management and revision of the manuscript. IL, DL, GD, S Corpus, PvD contributed to data collection and revision of the manuscript. S Chitturi, SE, MS, MH, CI, KD, MD, RL, JB, SS contributed intellectual input to the study design and revision of the manuscript. All authors were involved in revising the manuscript for important intellectual content and read and approved the final manuscript.

## Pre-publication history

The pre-publication history for this paper can be accessed here:

http://www.biomedcentral.com/1471-2393/13/221/prepub

## References

[B1] Australian Institute of Health and Welfare The health and welfare of Australia’s Aboriginal and Torres Strait Islander people, an overview 2011 2011 Canberra: AIHW: Cat no: IHW 42

[B2] Chamberlain C McNamara B Williams ED Yore D Oldenburg B Oats J Eades S Diabetes in pregnancy among indigenous women in Australia, Canada, New Zealand and the United States: a systematic review of the evidence for screening in early pregnancy Diabetes Metab Res Rev 2013 29 4 241 256 10.1002/dmrr.2389 23315909 PMC3698691

[B3] Porter C Skinner T Ellis I What is the impact of diabetes for Australian Aboriginal women when pregnant? Diabetes Res Clin Pract 2011 93 e29 e32 10.1016/j.diabres.2011.03.013 21481485

[B4] Australian Institute of Health and Welfare Diabetes in Pregnancy: its impact on Australian women and their babies. Diabetes series no.14 2010 Canberra: AIHW: Cat. no. CVD 52

[B5] Farrell T Neale L Cundy T Congenital anomalies in the offspring of women with type 1, type 2 and gestational diabetes Diabet Med 2002 19 4 322 326 10.1046/j.1464-5491.2002.00700.x 11943005

[B6] McElduff A Ross GP Lagstrom JA Champion B Flack JR Lau SM Moses RG Seneratne S McLean M Cheung NW Pregestational diabetes and pregnancy: an Australian experience Diabetes Care 2005 28 5 1260 1261 10.2337/diacare.28.5.1260 15855607

[B7] Bower C Stanley F Connell AF Gent CR Massey MS Birth defects in the infants of aboriginal and non-aboriginal mothers with diabetes in Western Australia Med J Aust 1992 156 8 520 524 1565042 10.5694/j.1326-5377.1992.tb121410.x

[B8] Metzger BE Lowe LP Dyer AR Trimble ER Chaovarindr U Coustan DR Hadden DR McCance DR Hod M McIntyre HD Hyperglycemia and adverse pregnancy outcomes N Engl J Med 2008 358 19 1991 2002 18463375 10.1056/NEJMoa0707943

[B9] Crowther CA Hiller JE Moss JR McPhee AJ Jeffries WS Robinson JS Effect of treatment of gestational diabetes mellitus on pregnancy outcomes N Engl J Med 2005 352 24 2477 2486 10.1056/NEJMoa042973 15951574

[B10] Bellamy L Casas JP Hingorani AD Williams D Type 2 diabetes mellitus after gestational diabetes: a systematic review and meta-analysis Lancet 2009 373 9677 1773 1779 10.1016/S0140-6736(09)60731-5 19465232

[B11] Mohamed N Dooley J Gestational diabetes and subsequent development of NIDDM in aboriginal women of Northwestern Ontario Int J Circumpolar Health 1998 57 Suppl 1 355 358 10093305

[B12] Fetita LS Sobngwi E Serradas P Calvo F Gautier JF Consequences of fetal exposure to maternal diabetes in offspring J Clin Endocrinol Metab 2006 91 10 3718 3724 10.1210/jc.2006-0624 16849402

[B13] Dabelea D Knowler WC Pettitt DJ Effect of diabetes in pregnancy on offspring: follow-up research in the Pima Indians J Matern Fetal Med 2000 9 1 83 88 10757442 10.1002/(SICI)1520-6661(200001/02)9:1<83::AID-MFM17>3.0.CO;2-O

[B14] Australian Bureau of Statistics Northern Territory at a Glance 2011 2011 Canberra: Bureau of Statistics, (ABS 1304.7)

[B15] Zhang X Dempsey KE Johnstone K Guthridge S Trends in the health of mothers and babies, Northern Territory: 1986–2005 2010 Darwin: Department of Health and Families

[B16] Martin FIR The diagnosis of gestational diabetes Med J Aust 1991 155 10 112 1857286

[B17] Nankervis A McIntyre HD Moses R Ross GP Callaway L Porter C Jeffries W Boorman C De Vries B McElduff A For the Australasian Diabetes in Pregnancy Society ADIPS Consensus Guidelines for the Testing and Diagnosis of Gestational Diabetes Mellitus in Australia 2013 http://www.adips.org/downloads/ADIPSConsensusGuidelinesGDM-03.05.13VersionACCEPTEDFINAL.pdf (Accessed 14 October 2013

[B18] Minymaku Kutju Tjukurpa Women’s Business Manual 2008 4 Alice Springs: Congress Alukura and Nganampa Health Council Inc

[B19] Metzger BE Gabbe SG Persson B Buchanan TA Catalano PA Damm P Dyer AR Leiva A Hod M Kitzmiler JL International association of diabetes and pregnancy study groups recommendations on the diagnosis and classification of hyperglycemia in pregnancy Diabetes Care 2010 33 3 676 682 20190296 10.2337/dc09-1848 PMC2827530

[B20] Cox JL Holden JM Sagovsky R Detection of postnatal depression. Development of the 10-item Edinburgh postnatal depression scale Br J Psychiatry 1987 150 782 786 10.1192/bjp.150.6.782 3651732

[B21] Brown ADH Mentha R Rowley KG Skinner T Davy C O’Dea K Depression in Aboriginal men in Central Australia: adaptation of the patient health questionnaire 9 BMC Psychiatry 2013 in press 10.1186/1471-244X-13-271 PMC3816593 24139186

[B22] The HAPO Study Cooperative Research Group Hyperglycemia and Adverse Pregnancy Outcome (HAPO) Study: Associations with Neonatal Anthropometrics Diabetes 2009 58 2 453 459 19011170 10.2337/db08-1112 PMC2628620

[B23] The HAPO Study Cooperative Research Group The Hyperglycemia and Adverse Pregnancy Outcome (HAPO) study Int J Gynecol Obstet 2002 78 1 69 77 10.1016/s0020-7292(02)00092-9 12113977

[B24] Catalano PM Thomas AJ Avallone DA Amini SB Anthropometric estimation of neonatal body composition Am J Obstet Gynecol 1995 173 4 1176 1181 10.1016/0002-9378(95)91348-3 7485315

[B25] Catalano PM Thomas A Huston-Presley L Amini SB Increased fetal adiposity: a very sensitive marker of abnormal *in utero* development Am J Obstet Gynecol 2003 189 6 1698 1704 10.1016/S0002-9378(03)00828-7 14710101

[B26] Hoy WE Nicol JL Birthweight and natural deaths in a remote Australian Aboriginal community Med J Aust 2010 192 1 14 20047542 10.5694/j.1326-5377.2010.tb03394.x

[B27] Yajnik CS Fall CH Coyaji KJ Hirve SS Rao S Barker DJ Joglekar C Kellingray S Neonatal anthropometry: the thin-fat Indian baby. The pune maternal nutrition study Int J Obes Relat Metab Disord 2003 27 2 173 180 10.1038/sj.ijo.802219 12586996

[B28] Landon MB Spong CY Thom E Carpenter MW Ramin SM Casey B Wapner RJ Varner MW Rouse DJ Thorp JM Jr A multicenter, randomized trial of treatment for mild gestational diabetes N Engl J Med 2009 361 14 1339 1348 10.1056/NEJMoa0902430 19797280 PMC2804874

[B29] Dabelea D Hanson RL Lindsay RS Pettitt DJ Imperatore G Gabir MM Roumain J Bennett PH Knowler WC Intrauterine exposure to diabetes conveys risks for type 2 diabetes and obesity: a study of discordant sibships Diabetes 2000 49 12 2208 2211 10.2337/diabetes.49.12.2208 11118027

[B30] Dabelea D Pettitt DJ Intrauterine diabetic environment confers risks for type 2 diabetes mellitus and obesity in the offspring, in addition to genetic susceptibility J Pediatr Endocrinol Metab 2001 14 8 1085 1091 11592564 10.1515/jpem-2001-0803

[B31] Gillman MW Oakey H Baghurst PA Volkmer RE Robinson JS Crowther CA Effect of treatment of gestational diabetes mellitus on obesity in the next generation Diabetes Care 2010 33 5 964 968 10.2337/dc09-1810 20150300 PMC2858199

[B32] Kelstrup L Damm P Mathiesen ER Hansen T Vaag AA Pedersen O Clausen TD Insulin resistance and impaired pancreatic beta-cell function in adult offspring of women with diabetes in pregnancy J Clin Endocrinol Metab 2013 98 9 3793 3801 10.1210/jc.2013-1536 23796568 PMC3763979

